# W/O/W Multiple Emulsified Microcapsules Based on Biopolymer Soybean Isolate Proteins: Improving Tannic Acid’s Biocompatibility and Sustained-Release Performance

**DOI:** 10.3390/molecules30112373

**Published:** 2025-05-29

**Authors:** Suning Zhang, Ruman Yan, Siyu Zhang, Yina Lu

**Affiliations:** 1 School of Perfume and Aroma Technology, Shanghai Institute of Technology, Shanghai 201418, China; siyu_zhang25@163.com; 2 Engineering Research Center of Perfume & Aroma and Cosmetics, Ministry of Education, Shanghai 201418, China; 3 JAKA Biotechnology Co., Ltd., Shanghai 201500, China; inna_lu@jakabiotech.com

**Keywords:** tannic acid, multiple emulsification, biocompatibility, microcapsule, soybean isolate proteins

## Abstract

Tannic acid (TA) possesses antioxidant, anticancer, and antibacterial properties. However, its pH sensitivity, protein cross-linking properties, and susceptibility to oxidation restrict its application. To address these challenges, W/O/W multiple emulsified TA microcapsules were developed using soybean protein isolate (SPI) as the natural wall material emulsifier through a two-step emulsification and spray drying process. The encapsulation efficiency of the obtained TA microcapsules was 87.6%, and TA’s thermal stability was significantly improved. TA microcapsules effectively reduced the acidity and irritability of TA, eliminated protein flocculation, and enhanced biocompatibility. Notably, the cell viability of the TA microcapsule (>94%) was significantly higher than free TA (65.6%). The storage stability test revealed that the microcapsules maintained structural integrity, with a retention rate of 96% after 10 days of storage. In vitro release studies of TA microcapsules demonstrated a sustained-release effect within 24 h. Simulated digestion studies further elucidated the protective effect of microcapsules on TA during gastric digestion. These multi-structured microcapsules based on SPI effectively address the limitations associated with TA utilization and enhance its potential for dual oral/transdermal administration in biomedical and cosmetic applications.

## 1. Introduction

Tannic acid (TA), a natural polyphenol with abundant hydroxyl and carbonyl groups, has emerged as a multifunctional agent in biomedical applications due to its antioxidant, anticancer, and antibacterial properties [1,2,3]. In contrast to synthetic antioxidants [4], TA exhibits superior bioactivity and has been extensively investigated in drug delivery and wound healing systems [5,6,7]. Notably, its dual functionality as both a therapeutic agent and a pharmaceutical excipient [8,9] underscores its unique value in pharmaceutical development. However, practical applications encounter significant challenges: The inherent astringency, pH sensitivity, and oxidation sensitivity of TA often compromise the stability of applied products and cause tissue irritation [10].

To enhance the antioxidant, antibacterial performance, stability, and system compatibility of TA, researchers have explored various natural and synthetic materials as carriers [11] to load TA, and prepared microcapsules, hydrogels and other product forms. Natural carriers such as hyaluronic acid, alginate, gelatin, and chitosan have demonstrated promising potential. For instance, chitin-based TA hydrogels [12] and chitosan-based TA microparticles [13] have shown enhanced antibacterial efficacy, while guar gum nanofiber-encapsulated TA significantly improved antioxidant capacity [14]. The core–shell structured nanoparticles with zein composite anionic pectin as the wall material can also prevent the flocculation of TA [15]. The composite of ovalbumin (OVA) and sodium alginate as the wall material can be utilized for the targeted delivery of active substances such as TA [16]. TA-loaded alginate microspheres can effectively prolong the antibacterial duration [17]. Among synthetic materials, including polyamide-6/hydroxyethyl cellulose nanofibers [18] and poly(methacrylic acid) nanoparticles [19], not only enhanced the coagulation capacity, but also achieved controlled release. However, current encapsulation strategies for active substances encounter limitations. In transdermal delivery systems, TA may be abruptly released from the mononuclear system, resulting in uneven distribution and skin irritation. The biosafety risks associated with synthetic carriers [20] and the potential damage to the skin barrier due to pH changes remain unresolved. Oral formulations are constrained by TA’s acidic instability in the gastric environment, necessitating improved bioavailability [21,22]. Consequently, the present study endeavors to develop encapsulation methods that synergistically enhance stability, optimize biocompatibility, and implement slow-release mechanisms.

Microencapsulation technology has garnered significant attention due to its exceptional encapsulation capabilities. Biopolymers, such as polysaccharides [23], proteins [24,25], and other wall materials [26,27], have garnered interest from the scientific community as matrices for microencapsulation, immobilization, or controlled release of diverse active compounds. These applications have been expanded across the pharmaceutical, food, biomedical, chemical, cosmetic, and textile industries, demonstrating promising outcomes. However, traditional single emulsions can only protect a single polar component. The water-soluble active ingredients may be free in the outer aqueous phase, and there is a possibility of combining with the wall material [28]. To address the aforementioned challenges, we have developed a two-step emulsification method to prepare W/O/W-type multi-compartmental microcapsules using soybean protein isolate (SPI) as a biopolymeric matrix. In contrast to conventional encapsulation systems, this method offers several distinct advantages. As a natural polymeric emulsifier with amphiphilic properties, SPI’s unique macromolecular structure enables effective emulsion stabilization without the need for organic solvents. This biopolymer provides sustainable and low-cost advantages [29,30]. Through the synergistic interaction between the physical shielding effect of the oil-phase matrix and the spray-drying technology, a dual-barrier mechanism is established. Notably, during the spray-drying process, the wall material formed by the evaporation of water in the external water phase cooperates with the retained oil phase core to effectively prevent the leakage of TA [31]. In contrast to intricate protein modification strategies, our method preserves the native functionality of SPI, thereby significantly simplifying the preparation process [32,33].

This study innovatively fabricated W/O/W multi-emulsion TA microcapsules using SPI as a natural wall material and emulsifier, without the use of organic solvents. The spray-drying technique was employed to prepare TA microcapsule powders with enhanced stability. The microcapsules were systematically characterized for morphology, structure, particle size distribution, functional groups, and thermal stability. System compatibility and irritability were evaluated through pH analysis, the hen’s egg test on the chorioallantoic membrane (HET CAM test), and a short-time exposure in vitro test method (STE). In vitro release behavior experiment and simulated digestion experiments elucidated release behaviors, demonstrating the system’s environmental friendliness, biosafety, and efficacy. This work provides a novel strategy for encapsulating hydrophilic polyphenols using sustainable protein matrices, offering insights for advanced delivery system design.

## 2. Results and Discussion

### 2.1. Effect of the Volume Ratio Between the Outer Water Phase (W_2_) and the W_1_/O Emulsion on the Multiple Structure and Stability of TA Composite Emulsion

**Figure 1:**
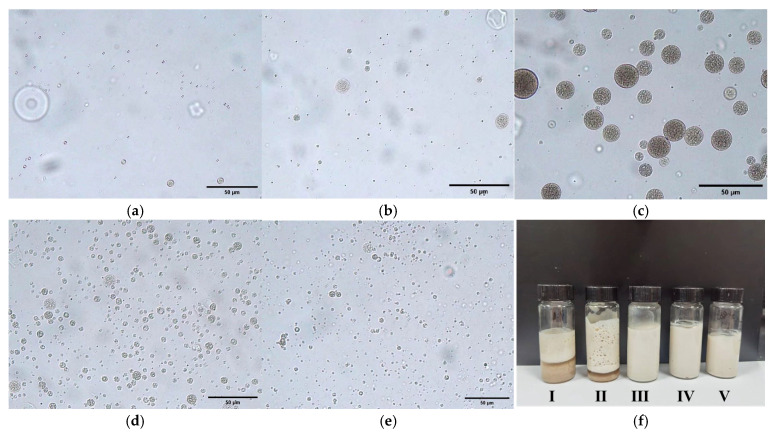
Optical microscope images of microcapsules prepared with varying outer water phase (W_2_) and W_1_/O emulsion ratios (200×) are presented: (**a**) 9:1; (**b**) 4:1; (**c**) 7:3; (**d**) 3:2; (**e**) 1:1; and (**f**) is the photograph of the corresponding macroscopic states of their emulsions.

The experimental results are shown in Figure 1 and Table 1. As the water content in the outer phase increased, the size of the emulsion particles initially increased and subsequently decreased. Multiple emulsions with outer water phase (W_2_) and W_1_/O emulsion ratios of 9:1 and 4:1 showed layering and demulsification. Samples with higher water content had more obvious layering (Figure 1f), but multiple emulsified particles can still be seen in the emulsified part after layering (Figure 1a). The multiple structures of emulsified particles with outer water phase (W_2_) and W_1_/O emulsion ratios of 7:3, 3:2, and 1:1 appeared regular and stable. The optical microscope images in Figure 1c–e showed that the microcapsules had a W/O/W-type multiple-emulsion structure, with relatively uniform particle sizes. Given the high encapsulation efficiency of water-soluble active substances, sample (III) with a lower oil phase content was selected for encapsulation. Consequently, the optimal outer water phase (W_2_) and W_1_/O emulsion ratio for the emulsion utilized in this experiment were determined to be 7:3. Additionally, the encapsulation efficiency of TA was calculated to approximately 87.6% using the methodologies outlined in Section 3.4.

### 2.2. Internal Structure, Surface Morphology, and Particle Size Distribution of Microcapsules

**Figure 2:**
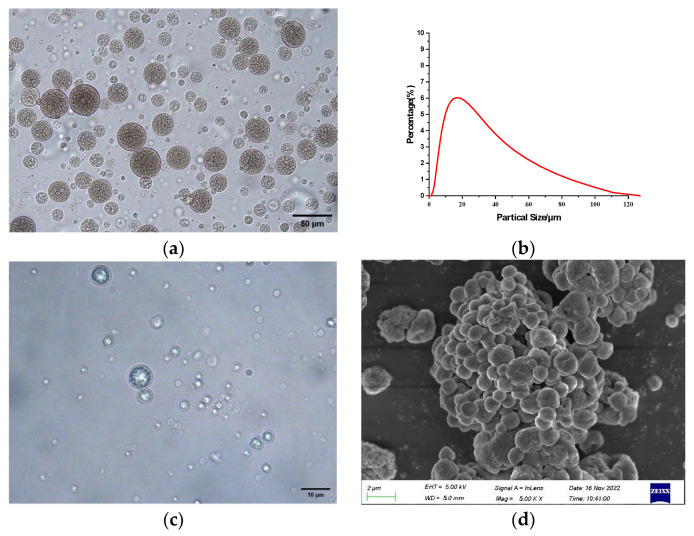
(**a**) 200× optical microscope image of TA microcapsules (before drying); (**b**) particle size distribution of TA microcapsules (before drying); (**c**) 500× optical microscope image of TA microcapsules (after drying); (**d**) 5.00 k× SEM image of TA microcapsules (after drying).

As shown in Figure 2a, the optical microscope image with a magnification of 200× revealed that the emulsion structure exhibited a distinct W/O/W multiple spherical structure, and the measured particle size distribution was shown in Figure 2b, with an average particle size of 21.0 μm. As can be seen from Figure 2c, in the optical microscope image magnified 500×, the TA microcapsule powder was spherical and had a distinct core–shell structure. Due to the loss of water in the water phase, the particle size was significantly reduced to 1–10 μm and the external morphology in Figure 2d showed that the surface of the dried microcapsules was relatively smooth, the wall structure was intact, and there were no cracks or holes, indicating that the microcapsules provide a good protective effect on the core material (TA).

### 2.3. Fourier Transform Infrared (FTIR) Spectroscopy

**Figure 3:**
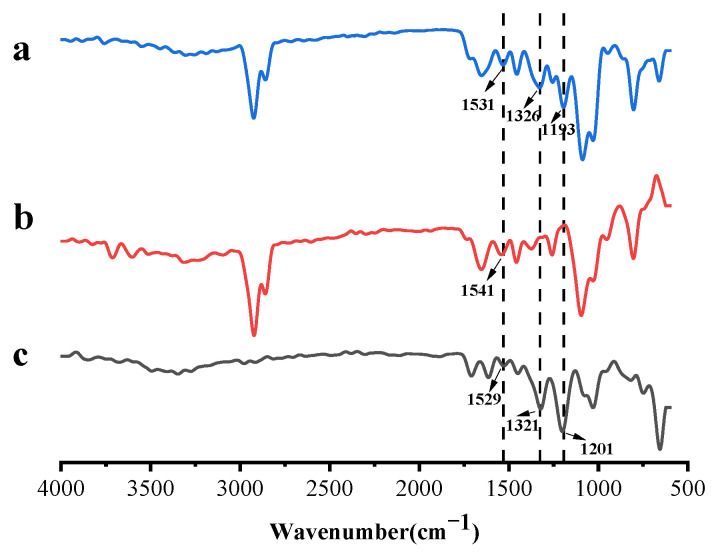
Infrared spectra of TA microcapsules (**a**), blank microcapsules (**b**), and TA (**c**).

Figure 3 was the FTIR spectra of TA microcapsules, TA, and blank microcapsules. In the TA spectrum, the broadband from 3600 cm^−1^ to 3000 cm^−1^ belonged to the -OH stretching vibration; the absorption peaks at 1614 cm ^−1^ and 1529 cm^−1^ were related to the -C-C- group in the aromatic ring. The absorption band at 1450 cm^−1^ was attributed to the deformation vibration of -C-C- in phenols; the absorption peak at 1321 cm^−1^ was the characteristic peak of the phenol group, and the band at 1201 cm^−1^ was the characteristic peak of the C-H bond; the absorption band at 1029 cm^−1^ was the C-O and C-H deformations [34,35,36]. For the blank microcapsules, the characteristic peak appearing at 1652 cm^−1^ was the absorption peak of the amide I band of SPI corresponding to the C-O stretching vibration. The characteristic peak appearing at 1541 cm^−1^ was the amide II band of SPI and corresponded to the N-H stretching vibration. The characteristic peak appearing at 1259 cm^−1^ was the C-N stretching vibration of the amide III band of SPI [37]. For TA microcapsules, it showed the characteristic peaks of both TA and blank microcapsules, and the absorption peak of the amide II band of SPI appeared at 1531 cm^−1^, compared with the blank microcapsules (1541 cm^−1^). The shift may be due to the vibration of the benzene ring skeleton of tannic acid at 1529 cm^−1^ affecting SPI [38]. The characteristic peak of the phenol group of TA appeared in the TA microcapsules at 1326 cm^−1^, and the characteristic peak of C-H of TA appeared at 1193 cm^−1^, indicating successful encapsulation of TA.

### 2.4. Thermal Stability Analysis

**Figure 4:**
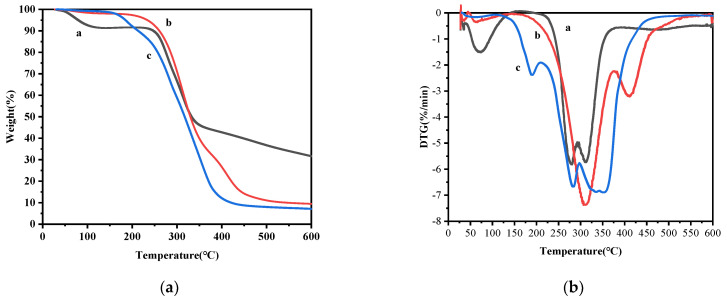
(**a**) Thermogravimetric curves of a: TA, b: blank microcapsules, and c: TA microcapsules; (**b**) DTG curves of a: TA, b: blank microcapsules, and c: TA microcapsules.

Figure 4a,b were the thermogravimetric (TG) and derivative thermogravimetric (DTG) curves of TA, blank microcapsules and TA microcapsules, respectively. The thermal decomposition temperatures of each sample at different stages were presented in Table 2. It can be seen from the thermogravimetric curve that TA had two stages of weight loss. The initial stage was between 30 °C and 130 °C, with a weight loss of about 8%. The weight loss rate attained its peak at 75 °C, primarily attributed to the evaporation of water absorbed by TA. The subsequent stage was from 200 °C to 375 °C, with the highest weight loss rates at 280 °C and 310 °C, respectively. The weight loss rate in this stage was predominantly caused by the release of CO_2_ as TA gradually decomposed into pyrogallic acid upon heating to 210~215 °C, accompanied by the volatilization of pyrogallic acid (boiling point 309 °C) [39]. The weight loss of blank microcapsules was about 2% at temperatures between 50 °C and 150 °C and reached 60% at temperatures between 200 °C and 360 °C. This can be attributed to the volatilization of certain oil-phase components present in the microcapsule powder. Concurrently, the wall SPI underwent decomposition at around 250 °C and reached its maximum weight loss rate at 310 °C [40,41]. When the temperature reached 525 °C, about 10% of the weight remained. In contrast, the mass loss of TA microcapsules was characterized by four distinct stages. The initial stage (50~150 °C) resulted in a negligible weight loss of 2%. This can be attributed to the loss of bound water within the microcapsules. The second stage (150–250 °C) witnessed a weight loss of approximately 15%. Notably, the maximum weight loss rate occurred at 175 °C, as compared with curves b and c in Figure 4b. This stage was primarily attributed to the evaporation of water from encapsulated TA, leading to a significant increase in the weight loss temperature. The evaporation temperature of the inner water phase after encapsulation was consequently elevated. The third stage (250–500 °C) resulted in a total weight loss of 80%. The maximum loss rates were observed at 288 °C and 350 °C, respectively. This stage was characterized by the simultaneous degradation of both the TA and SPI. Finally, about 10% of the mass remained at 500 °C.

A comparative analysis of the mass loss rates of TA microcapsules and TA from the DTG curves reveals that the peak mass loss rate of TA occurred at 280 °C and 310 °C. Conversely, the maximum weight loss rate of TA microcapsules exhibited a significant increase at 288 °C and 350 °C. Within the temperature range of 260 °C to 400 °C, the mass loss rate of TA microcapsules exhibited a pronounced superiority over both TA and blank microcapsules. This observation suggests that during this temperature interval, not only the external phase material of TA microcapsules, but also the TA encapsulated within the microcapsules underwent decomposition. This phenomenon indicates that the microencapsulation of TA enhances its thermal stability to some extent. In summary, the encapsulation of TA within microcapsules results in a slower decomposition rate due to the protective effect of multiple structures and SPI, thereby contributing to an improved thermal stability.

### 2.5. Effect of TA Microcapsules on Solution pH

**Figure 5:**
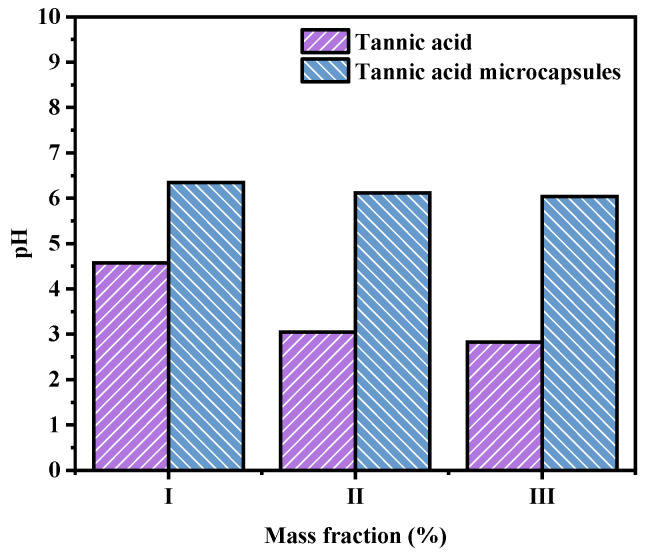
Different concentrations of tannic acid and microcapsules containing corresponding concentrations of tannic acid were subjected to a pH test. I: 1% tannic acid and microcapsules containing 1% tannic acid, II: 5% tannic acid and microcapsules containing 5% tannic acid, III: 10% tannic acid, and microcapsules containing 10% tannic acid.

As can be seen from Figure 5, the pH values of 1%, 5%, and 10% TA solutions were 4.57, 3.05, and 2.83, respectively. In contrast, the pH values of microcapsule solutions containing 1%, 5%, and 10% TA were 6.35, 6.12, and 6.04, respectively. It can be seen that the encapsulation of TA by microcapsules facilitates the reduction in acidity of TA in the environment. This enables the application of TA to various products without affecting the pH of the product environment.

### 2.6. The HET-CAM Test of TA and TA Microcapsules

Non-covalent interactions, such as hydrophobic interactions or hydrogen bonds, may occur between TA and proteins [42]. TA can also be covalently bound to proteins after being converted into quinone or phenol forms [43,44]. Furthermore, TA exhibits semi-colloidal properties in aqueous solutions. When TA molecules dissociate in solution, the negative charges in the TA aqueous solution generate electrokinetic potential. TA is a natural polymer substance with a large volume and is easy to flocculate and precipitate from water [45]. At high concentrations of TA, “sequential binding” occurs between TA and proteins; that is, the initial binding of TA at specific sites on the protein will lead to conformational changes, thereby increasing the binding affinity of TA and the protein and causing precipitation [46].

**Figure 6:**
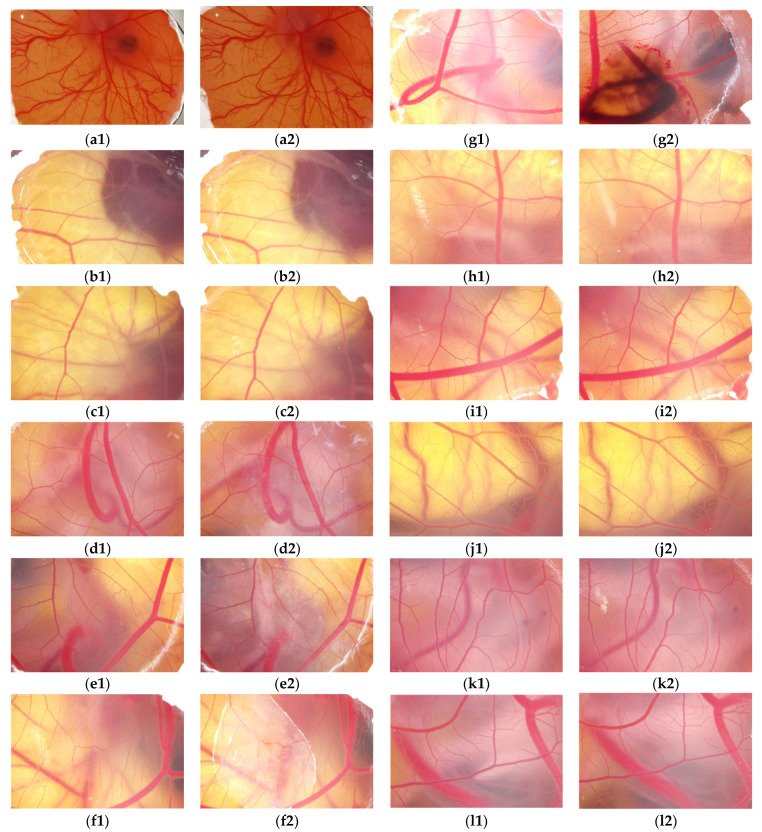
The irritating effects of positive, negative control, TA microcapsules and the corresponding amount of TA solution on the CAM. (**a1,a2**) were CAM images before and after negative control saline treatment, respectively; (**b1**,**b2**) were CAM images before and after 0.5% TA treatment, respectively; (**c1**,**c2**) were CAM images before and after 1% TA treatment, respectively; (**d1**,**d2**) were CAM images before and after 2% TA treatment, respectively; (**e1**,**e2**) were CAM images before and after 5% TA treatment, respectively; (**f1**,**f2**) were CAM images before and after 10% TA treatment, respectively; (**g1**,**g2**) were CAM images before and after positive control saline treatment, respectively; (**h1**,**h2**) were CAM images before and after treatment with microcapsules containing 0.5% TA, respectively; (**i1**,**i2**) were CAM images before and after treatment with microcapsules containing 1% TA, respectively; (**j1**,**j2**) were CAM images before and after treatment with microcapsules containing 2% TA, respectively; (**k1**,**k2**) were CAM images before and after treatment with microcapsules containing 5% TA, respectively; (**l1**,**l2**) were CAM images before and after treatment with microcapsules containing 10% TA, respectively.

The effects of TA and microcapsules with equal amounts of TA content on blood vessels and proteins of the CAM were shown in Figure 6. The expression of Figure 6(a1,a2) showed that the negative control sample had no significant effect on the CAM blood vessels and egg white protein of the CAM, while Figure 6(g1,g2) indicated that obvious bleeding occurred in the CAM after treatment with the positive control product, but no protein denaturation occurred. Figure 6(b1–c2) indicated that 0.5% and 1% TA solutions had no noticeable effect on the CAM, and no bleeding, coagulation, hemolysis, or protein flocculation was observed. However, Figure 6(d1–f2) showed that 2%, 5%, and 10% TA solutions had a significant impact on the CAM, resulting in the appearance of white flocculent material, which was attributed to TA at a higher concentration, can bind to ovalbumin and induce protein flocculation. By contrast, Figure 6(h1–l2) showed that the microcapsule dispersion encapsulating the corresponding amount of TA did not have any adverse effects on the CAM, such as bleeding, coagulation, hemolysis, and white flocculent substances, indicating that the encapsulated TA had no significant effect on the CAM. Therefore, the protein flocculation caused by TA disappeared or was weakened. TA microcapsules could reduce the impact of TA on proteins and other components of the system to a certain extent, and enhance biocompatibility during use.

### 2.7. The STE Test of TA and TA Microcapsules

As shown in Table 3, low-concentration TA solutions had little effect on rabbit corneal epithelial cells. However, as the TA concentration increased, the cell viability of these cells progressively declined. The cell viability of SIRC cells exposed to 0.5% TA could still reach 97.74%, while the viability of cells exposed to 2.5% and 5% TA was reduced to 83.86% and 65.59%, respectively. In comparison, the cell viability of SIRC cells exposed to TA microcapsules containing 0.5% and 2.5% TA was as high as 100.63% and 101.55%, even for those exposed to TA microcapsules containing 5% TA, their survival rate still reached 94.65%. As TA concentration increased, the pH value decreased, and acidity increased. Additionally, its cross-linking and flocculation effects on cell proteins were enhanced, thereby impacting cell survival. The encapsulation of TA within microcapsules effectively mitigated the acidic stimulation of TA within the solution. As previously mentioned, the pH value was generally maintained at around 6.0, which falls within the slightly acidic range. Furthermore, the direct interaction between TA and cells was thereby avoided, significantly enhancing the biocompatibility between TA and cells.

### 2.8. Storage Stability of Microcapsules

**Figure 7:**
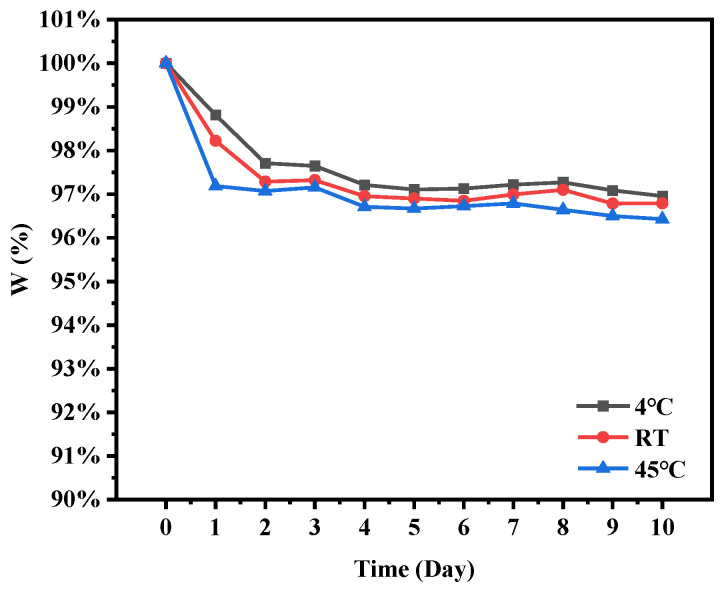
Retention rate of TA microcapsules at different temperatures.

As shown in Figure 7, the storage stability of TA microcapsules exhibited remarkable resilience. After a 10-day storage stability testing period, TA retention rate of the microcapsules consistently exceeded above 96%. The retention of TA in TA microcapsules experienced a slight decline during the initial three days of storage at 4 °C, room temperature (RT), and 45 °C. However, after this initial period, the TA retention rate remained relatively stable, affirming the effectiveness of the microcapsules in protecting TA during long-term storage durations. Furthermore, the lower temperatures (4 °C) proved to be more favorable for the storage of TA microcapsules. As a biodegradable substrate, SPI showed excellent performance in protecting and preserving TA, thus enhancing its storage stability and further validating the structural stability of the prepared microcapsules.

### 2.9. In Vitro Release Behavior Experiment

**Figure 8:**
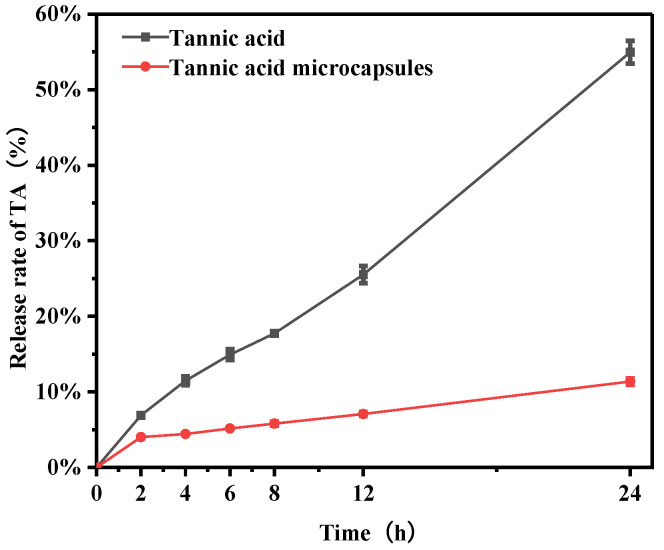
Experimental results of in vitro release behavior of tannic acid and tannic acid microcapsules.

PBS buffer was used as the receiving solution, and samples were taken at regular intervals within 24 h to measure the UV absorbance at 278 nm to investigate the release behavior of TA and TA microcapsules. As shown in Figure 8, TA dissolved in water and exhibited a rapid release rate, with approximately 55% of the TA released within 24 h. The remaining unreleased TA may be attributed to its active phenolic groups, which could potentially form poly (tannic acid), thereby extending the time it takes to pass through the semipermeable membrane [47]. In contrast to TA, the release rate of TA in TA microcapsules was significantly slower, with only about 10% of TA released within 24 h. In contrast to the limited release of TA in the Inês Guimarães study [13] (0.77 ± 0.003% TA release after 24 h) and the pre-sudden release of mCSB with TA [17] microspheres (58.89% release in the first 16 h), this observation suggests that TA microcapsules possess sustained-release properties.

### 2.10. In Vitro Simulated Digestion and Release of TA Microcapsules

**Figure 9:**
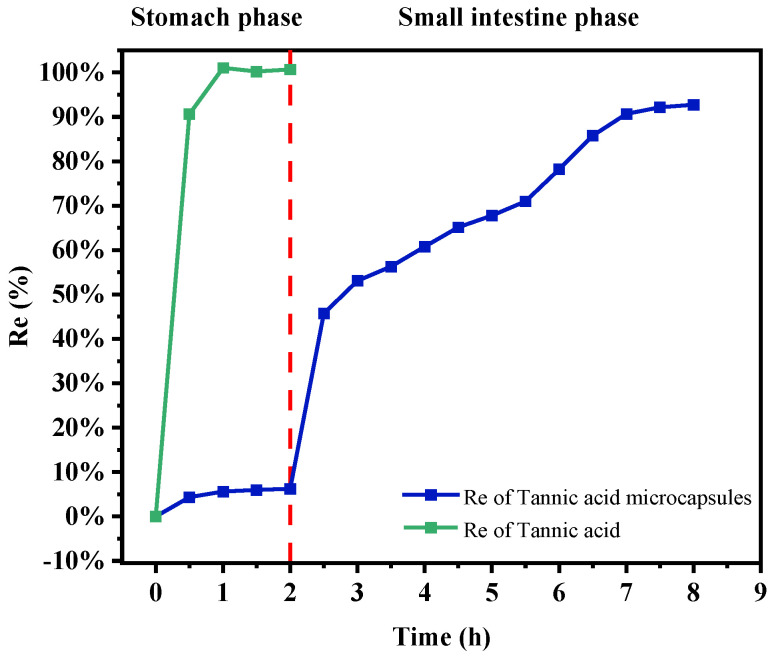
Percentage of TA released during incubation of tannic acid and TA microcapsules in simulated gastric and small intestinal fluids.

The TA release curves of TA and TA microcapsules under simulated gastric and small intestinal conditions are shown in Figure 9. Free TA was rapidly released into gastric juice, with 90.61% of TA released after 30 min and 100% released after 60 min. This finding is consistent with the results reported by X. Liang et al. [15,48]. In contrast, the release rate of TA in microcapsules was significantly slower, with only approximately 6.22% of TA released into gastric juice after 120 min of digestion. This value is substantially lower than the 31% TA release reported by Xiao Liang for the identical gastric digestion period. The slower release of encapsulated TA may be attributed to the incomplete hydrolysis of the SPI of microcapsules by pepsin, thereby protecting TA in the gastric juice environment [49]. Once the microcapsules reached the small intestinal stage, within the initial 30 min of small intestinal digestion, the environment around the microcapsules underwent a change, which facilitated the gradual hydrolysis of SPI by trypsin, leading to a substantial increase in the TA release rate. Subsequently, TA was gradually released within 6 h after digestion in the small intestine. These findings suggest that TA microcapsules can effectively retain a substantial portion of TA in the gastric fluid environment, facilitating its release into the small intestine. This process potentially protects polyphenols from degradation in the stomach while maintaining their bioaccessibility.

### 2.11. DPPH Radical Scavenging Assay

**Figure 10:**
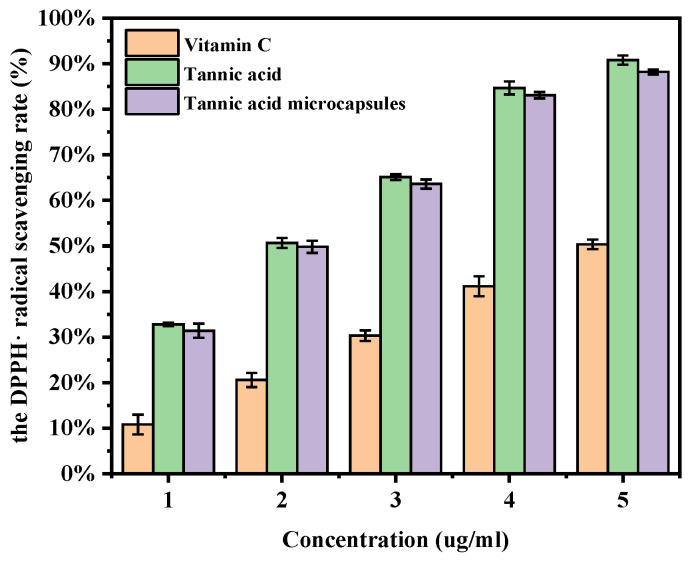
The DPPH· radical scavenging activity of VC, TA and TA microcapsules at various concentrations.

As depicted in Figure 10, the results of the experiment demonstrated a notable increase in the DPPH· radical scavenging rate of VC as the concentration was elevated from 1 μg/mL to 5 μg/mL, reaching a maximum of 50.35%. In contrast, the DPPH· radical scavenging rate of the TA aqueous solution exhibited a substantial surge, reaching a peak of 90.78% at the same concentration. Furthermore, the DPPH· radical scavenging rate of TA microcapsules also demonstrated a significant enhancement, reaching a maximum of 88.17% at the same concentration. Among these three formulations, the antioxidant activities of TA and TA microcapsules were found to be significantly superior to those of VC at the same concentration. Notably, under the same concentration gradient, there was no discernible difference in the scavenging effect between TA and TA microcapsules. This outcome underscores the fact that microencapsulation treatment of TA did not result in a significant reduction in its antioxidant capacity. Consequently, TA microcapsules retained their stable and efficient free radical scavenging performance, providing strong support for the practical application of TA in the field of antioxidation.

## 3. Materials and Methods

### 3.1. Experimental Materials

TA (≥98%) was provided free of charge by JAKA Biotechnology Co., Ltd. (Shanghai, China), liquid paraffin (CP) was purchased from Shanghai Boer Chemical Reagent Co., Ltd. (Shanghai, China), ABIL EM90 (cetyl polyethylene glycol/polypropylene glycol −10/1 dimethylsiloxanol, cosmetic grade) was purchased from Shanghai Baihaobo Biotechnology Co., Ltd. (Shanghai, China), Tween 20 (CP), 1, 3-butanediol (99%, RG), NaOH (≥96%, AR), NaCl (≥99.5%, AR) was purchased from Sinopharm Chemical Reagent Co., Ltd. (Beijing, China). Soybean protein isolate (BR), trypsin (from porcine pancreas) (BR,1;250), and pepsin (from pig gastric mucosa) (USP,1:3000) were purchased from Shanghai Yuanye Biotechnology and Limited Company (Shanghai, China). Deionized water was used in all experiments. These chemicals were used as received without further purification.

### 3.2. Preparation of Multiple Emulsified TA Microcapsules

**Figure 11:**
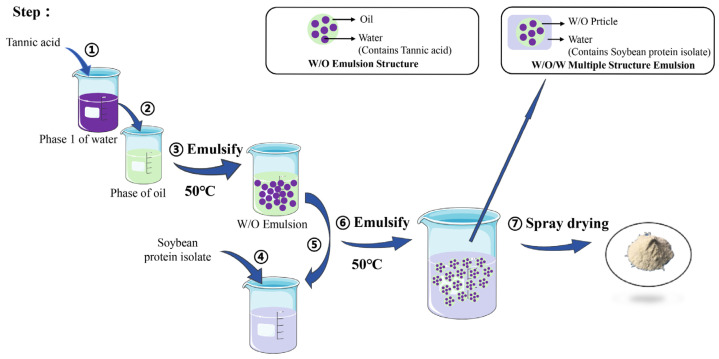
Preparation of TA microcapsules with multiple emulsified structure.

W/O/W multiple emulsion was prepared in two steps. First was the preparation of the W_1_/O emulsion: the internal water phase was created by dissolving TA (3%, *w/w*) in NaCl solution (0.5%, *w/w*) and stirring at 50 °C with a magnetic stirrer (Feb-90 Zhen Rong, Shanghai, China); the oil phase was obtained by adding emulsifier EM90 to liquid paraffin; then, W_1_ was dispersed into the oil phase (O:W_1_ is 3:7, *w/w*), and homogenized using a high-speed shear homogenizer (FA25, FLUKO, Shanghai, China) at 10,000 rpm for 3 min to obtain W_1_/O emulsion. The second step was the preparation of the composite emulsion: first, SPI (3%, *w/w*), Tween20 (2%, *w/w*), and 1,3-butanediol (10%, *w/w*) were dissolved in water at 40 °C with stirring to obtain the outer water phase (W_2_). Then, W_1_/O emulsion and the outer water phase (W_2_) were mixed at a ratio of 3:7 (wt%), and homogenized and emulsified evenly at 50 °C and 10,000 rpm for 3 min to obtain W_1_/O/W_2_ emulsion. Under the specified conditions of inlet air temperature at 165 °C, outlet air temperature at 80 °C, and feed rate of 1.5 mL/min, the W_1_/O/W_2_ emulsion was spray-dried using a spray dryer (Hefan, Shanghai, HF 015) to produce TA microcapsules. The preparation process is shown in Figure 11 below.

### 3.3. Effect of the Volume Ratio Between the Outer Water Phase (W_2_) and the W_1_/O Emulsion on the Multiple Structure and Stability of TA Composite Emulsion

In the W/O/W emulsion system, the composition of the external water phase significantly impacts the stability of the entire emulsion and the morphology of its particles [50,51]. To elucidate the influence of varying external water phase (W_2_) and W_1_/O emulsion ratios on emulsion stability, we prepared emulsions with distinct ratios (9:1, 4:1, 7:3, 3:2, and 1:1). The emulsion preparation methodology is detailed in Section 3.2. The prepared emulsions were subjected to macroscopic stability assessments and their particle morphology was observed using a microscope.

### 3.4. Determination of the Encapsulation Efficiency

The free TA on the surface of the microcapsules was eluted by centrifugation with anhydrous ethanol. The supernatant after centrifugation was analyzed using a UV-visible spectrophotometer (UV-2102. Unico, Shanghai, China) at the characteristic absorption peak of TA at 278 nm, and the amount of free TA was determined using a standard curve. Each measurement was repeated at least three times. The encapsulation efficiency (EE) of microcapsules was calculated according to the following equation:

EE (%) = (W core − W surface)/W core × 100%(1)

In equation: W _core_—the amount of TA invested in microcapsules; W _surface_—the amount of free TA content on the surface of microcapsules.

### 3.5. Internal Structure, Surface Morphology, and Particle Size Distribution of Microcapsules

The internal microstructure of the multiple-structure emulsion and microencapsulated powder was characterized using an optical microscope (CDM-806C, TM, China). The TA microcapsules powder was further adhered to the conductive resin, and the surface morphology was characterized with a scanning electron microscope (Hitachi SU8010, Tokyo, Japan) at an accelerating voltage of 15 kV.

The multiple-structure emulsion was diluted with deionized water and then added to the sample cell of a Zetasizer nanolaser particle size analyzer (Mastersizer 2000, Malvern Instruments Ltd., Malvern, Worcestershire, UK) to measure the diameter and size distribution of the microcapsules.

### 3.6. FTIR Characterization

At room temperature, TA, spray-dried blank microcapsules, and spray-dried TA microcapsules were characterized by Fourier infrared spectroscopy (Fourier Infrared Analyzer, Vertex70, Bruker, Germany) in the range of 500–4000 cm^−1^ wave number with a resolution of 4 cm^−1^, respectively.

### 3.7. Thermogravimetric Analysis (TGA)

The thermal stability of TA, blank microcapsules, and TA microcapsules was evaluated using a thermogravimetric analyzer (TGA) (TGA Q-5000, TA Instrument Co., Ltd. (New Castle, DE , USA)). All measurements were conducted in a N_2_ atmosphere at a flow rate of 20 mL/min, and the temperature range was scanned from room temperature to 600 °C at a heating rate of 10 °C/min [52,53].

### 3.8. Measurement of System pH Changes

A pH meter (Leici pH S-3C, INESA, Shanghai, China) was used to measure the pH values of TA solutions with mass fractions of 1%, 5%, and 10%, as well as TA microcapsule solutions containing equivalent TA mass fractions, respectively, to examine the changes in acidity of the system before and after TA encapsulation.

### 3.9. The HET-CAM Test

The HET-CAM assay is an in vitro eye irritation assessment method in the cosmetic and pharmaceutical fields that is widely used as an alternative to animal testing. The irritation of substances was assessed by monitoring changes in bleeding, coagulation, and vasolysis in the chorioallantoic membrane (CAM) [54,55]. Endpoint evaluation or response time evaluation is determined by the transparency of the test sample. Since the high-concentration TA solution exhibits a darker color and the microcapsule powder is opaque, the experiment was conducted using the endpoint evaluation method. Detailed detection steps are provided in reference [56].

In this experiment, a 0.1 mol/L sodium hydroxide solution was employed as a positive control, while a 0.9% sodium chloride solution served as a negative control. The endpoint evaluation method was utilized to assess the stimulatory effects of 0.5%, 1%, 2%, 5%, and 10% TA solutions, as well as TA microcapsule turbid dispersions with identical TA content, respectively. Given that the results of this experiment indicated that TA did not manifest any obvious changes in bleeding, coagulation, or vasolysis on the CAM, the flocculation phenomenon of chicken embryo proteins before and after TA encapsulation was the primary focus of attention while examining the blood vessels of chicken embryos.

### 3.10. Short Time Exposure In Vitro Test Method (STE)

Using the STE, rabbit corneal epithelial cells were exposed to chemical materials for a short period of time to simulate acute corneal irritation. The cell viability was calculated to predict eye injury caused by chemical materials. SIRC (Qingqi Biotech., Shanghai, China) cells were seeded into a 96-well plate at a density of 6000 cells/well with MEM complete medium containing 10% fetal bovine serum, 2 mM glutamine and 1% penicillin-streptomycin (Thermo Fisher, Waltham, MA, USA). A total of 200 μL phosphate-buffered saline (PBS) was added to the peripheral wells, and the plate was incubated in a CO_2_ incubator (Thermo Fisher, USA) at 37 °C, 5% CO_2_ for 4 days. After that, the supernatant was discarded, 200 μL of diluted sample or control were added to each well with at least three replicates. The MEM complete medium was used as a blank control, the cells cultured in complete medium were used as a negative control, 0.01% sodium lauryl sulfate (SLS, Sinopharm, China) saline solution was used as a positive control, and saline solution was used as a solvent control, TA solutions of 0.5%, 2.5%, and 5% and TA microencapsulated dispersion with the same TA content were used as experimental sample solutions. Treatment of cells was performed in a CO_2_ incubator for 5 min. After incubation, the medium was discarded and 200 μL of PBS was added to wash the cells twice. The absorbance value (OD_450_) at a wavelength of 450 nm was measured using a multifunctional microplate reader (Multiskan FC, Thermo Fisher, USA) with the CCK-8 staining method [57], using 10 μL CCK-8 solution (Viva cell, Shanghai Xiaopeng Biotechnology Co., Ltd., Shanghai, China) in 100 μL medium incubated at 37 °C for 1 h. The cell viability was calculated according to the following equation. The experiment was repeated 3 times, and the sample was judged based on the average relative cell viability of 3 tests.

Cell viability (%) = (OD_450 sample_ − OD_450 blank control_)/(OD_450 solvent control_ − OD_450 blank control_) × 100% (2)

In equation: OD_450 sample_—Test the absorbance value of the sample at 450 nm; OD_450 blank control_—absorbance value of the blank control at 450 nm; OD_450 solvent control_—absorbance value of solvent control at 450 nm.

### 3.11. Storage Stability of Microcapsules

The newly prepared TA microcapsule powder was sealed and stored at 4 °C, room temperature (RT), and 45 °C, respectively. A certain amount of TA microcapsule powder was dissolved in anhydrous ethanol each day for 10 days, and then centrifuged to elute the free core substance from the surface of the microcapsule. The absorbance of TA in the supernatant was measured using a UV spectrophotometer (UV-2102. Unico, Shanghai, China) at 278 nm to quantify the free core substance, and its encapsulation efficiency was calculated to determine the encapsulated core substance content. The core substance content within the initial encapsulation was set to be 100%, and the storage stability of TA microcapsules was evaluated by calculating the retention rate (W) according to the following equation.

W (%) = W_n_/W_0_ × 100% (3)

In equation: W_n_—nth day TA encapsulation content; W_0_—initial TA encapsulation content.

### 3.12. In Vitro Release Behavior Test

The release behavior of TA and TA microcapsules was evaluated using a Franz diffusion cell (Drug Transdermal Diffusion Tester, RJY-6B, Shanghai Huanghai Drug Testing Instrument Co., Ltd. (Shanghai, China)). The Franz diffusion cell uses a microporous filter to simulate the semi-permeable membrane of artificial skin, which can selectively allow specific substances to pass through and filter particles of specific sizes. A Millipore filter was positioned between the donor and receptor chambers of the Franz diffusion cell with an effective permeation area of 2.2 cm^2^ and a receiver cell volume of 6.5 mL. PBS buffer was used as the receptor solution and incubated at 37 ± 0.2 °C with stirring at 550 rpm in a water bath. The test samples were dissolved in deionized water at a concentration of 10 mg/mL and added to the donor chamber. Samples (0.2 mL) were withdrawn from the receptor chamber at predetermined time intervals and then replaced with an equal volume of fresh PBS buffer. The concentration of TA in the receptor compartment was calculated by diluting the 0.2 mL sample a specific number of times and measuring the concentration of TA in the diluted liquid at 278 nm using a UV spectrophotometer (UV-2102, Unico, Shanghai, China). This method can effectively evaluate in vitro release characteristics of TA and TA microcapsules.

### 3.13. In Vitro Simulated Digestion of Tannic Acid Microcapsules

The in vitro release behavior of TA microcapsules under the simulated gastrointestinal model was based on an existing method [58] with slight modifications. An amount of 1 g of pepsin was dissolved in 100 mL of NaCl (0.03 M), and the pH was then adjusted to 2.0 with 1.0 mol/L HCl to prepare simulated gastric juice. Simulated intestinal fluid was prepared by dissolving 1 g of trypsin in PBS solution (0.2 mol/L), and then adjusting the pH to 8.0 with 0.1 M NaOH. To evaluate the in vitro release of TA from microcapsule powder, 200 mg of microcapsule powder was added to 20 mL of simulated gastric fluid at pH 2.0, and the mixture was stirred at 100 rpm in a water bath at 37 °C for 2 h to simulate gastric digestion (0–2 h). During the digestion process, 2 mL of digestive juice was collected every 0.5 h, and an equal volume of fresh simulated gastric juice was added. After 2 h of digestion in gastric juice, 20 mL of simulated intestinal juice was added. The pH of the resulting solution was adjusted to 8.0, and digestion was conducted for a duration of 6 h. Digestive fluid (2 mL) was collected at 0.5 h intervals and subsequently replenished with equal volumes of simulated intestinal fluid. After the collected samples were centrifuged, the supernatant was extracted and the absorbance of TA was measured at 278 nm. The release amount was then calculated to determine the TA release rate (Re) in the microcapsule powder. The release rate was calculated using the following equation:

Re (%) = R/R_0_ × 100% (4)

In equation: R—TA released in the production solution; R_0_—TA content in the input microcapsules.

### 3.14. DPPH Radical Scavenging Assay

DPPH· radical scavenging assay was conducted using the method proposed by [59]. Anhydrous ethanol served as a blank control group. Vitamin C aqueous solution with effective concentrations of 1, 2, 3, 4, and 5 μg/mL of vitamin C were used as control groups, Similarly, TA aqueous solution with effective concentrations of 1, 2, 3, 4, and 5 μg/mL of TA, and TA microcapsule dispersions were prepared, respectively. At room temperature, 2 mL of 0.2 mM DPPH–ethanol solution and 2 mL of different concentrations of the sample solution were mixed homogeneously and reacted in the dark for 30 min. The absorbance of the solution was measured at 517 nm using a UV spectrophotometer. The DPPH· radical scavenging rate (%) was calculated using the following equation:

DPPH radical scavenging rate (%) = (A_0_ − A_1_)/A_0_ × 100% (5)

where A_0_ is the absorbance of the blank control group, while A_1_ is the absorbance of the sample solution.

A bar graph of the DPPH· radical scavenging activity was plotted with the reaction concentration (ug/mL) as the *X*-axis and the DPPH· radical scavenging rate (%) as the *Y*-axis.

## 4. Conclusions

The encapsulation of water-soluble active ingredients and their stability in aqueous formulations remain challenging in the field of controlled release. In this study, TA microcapsules with a W/O/W multiple emulsion structure were successfully fabricated using SPI as a natural wall material through a green, solvent-free process. The optimized microcapsules exhibited an encapsulation efficiency of 87.6%, an average particle size of 21 μm, and retained 96% of TA after 10-day storage. Thermogravimetric analysis confirmed enhanced thermal stability. The preparation method avoids the use of organic solvents, is both environmentally friendly and biocompatible, and addresses the challenges of the encapsulation of water-soluble active ingredients and stabilizing them in aqueous formulations. The results showed that the TA microcapsules could neutralize the acidity of the formulation, and the solution pH was raised from acidic 2.83 to neutral 6.04 when the effective concentration of TA was 10%. The HET-CAM test indicated that when the effective concentration of TA was ≥2%, TA microcapsules eliminated the protein flocculation caused by TA. The STE test revealed 94.65% cell viability retention at 5% effective TA concentration, demonstrating a significant reduction in biological system stimulation. In the simulated gastric environment, the release of TA within 2 h was ≤6.22%, and in the subsequent simulated intestinal environment, the release reached 82% within 6 h, achieving controllable release in the gastrointestinal tract. The microcapsules showed a continuous, slow release over 24 h. The release of TA from the microcapsules was 10% at 24 h, which was significantly lower than the 55% release of free TA. TA microcapsules still maintained good antioxidant activity. These findings provide new insights into the protection and slow release of water-sensitive components, as well as new perspectives on the potential applications of oral and transdermal delivery systems.

## Figures and Tables

**Table 1:** Structural stability of TA multiple emulsions prepared with different outer water- phase (W_2_) and W_1_/O emulsion ratios.

Multiple Emulsions	I	II	III	IV	V
Ratio of outer water phase (W_2_) and W_1_/O emulsion	9:1	4:1	7:3	3:2	1:1
Stability	layered, broken	layered, broken	stable	stable	stable

**Table 2:** Thermal decomposition parameters obtained through TG/DTG analysis.

Sample	Decomposition Stage	Tonset (°C)	Tpeak (°C)
TA	30–130 °C	30	75
	200–375 °C	200	280–310
Blank microcapsules	50–150 °C	50	-
	200–360 °C	200	310
	360–500 °C	360	408
TA microcapsules	50–150 °C	50	-
	150–250 °C	150	175
	250–500 °C	250	288–350

**Table 3:** In vitro experimental data on cell viability of SIRC cells using STE to different concentrations of TA and microcapsules encapsulating corresponding concentrations of TA.

Group	Concentration (%)	STE: Cell Viability (%)			
		First	Second	Third	Average ± SD
Negative control	-	104.53	104.95	102.97	104.15 ± 1.05
Positive control	-	57.22	58.42	64.05	59.90 ± 3.65
TA	5	62.09	65.87	68.82	65.59 ± 3.37
	2.5	78.33	84.81	88.44	83.86 ± 5.12
	0.5	99.71	83.49	110.01	97.74 ± 13.37
TA microcapsules corresponding amount of TA	5	94.95	95.53	93.46	94.65 ± 1.07
	2.5	104.63	97.33	102.69	101.55 ± 3.78
	0.5	102.58	100.38	98.92	100.63 ± 1.84

SD, standard deviation; STE, short time exposure (test).

## Data Availability

Data are contained within the article.

## References

[1] Baldwin A., Booth B.W. (2022). Biomedical applications of tannic acid. J. Biomater. Appl..

[2] Nakamura T., Yoshida N., Yasoshima M., Kojima Y. (2017). Effect of tannic acid on skin barrier function. Exp. Dermatol..

[3] Bouki E., Dimitriadis V.K., Kaloyianni M., Dailianis S. (2013). Antioxidant and pro-oxidant challenge of tannic acid in mussel hemocytes exposed to cadmium. Mar. Environ. Res..

[4] Fu X., Yuan S., Yang F., Yu H., Xie Y., Guo Y., Yao W. (2023). Characterization of the interaction between boscalid and tannic acid and its effect on the antioxidant properties of tannic acid. J. Food Sci..

[5] Chen Y., Tian L., Yang F., Tong W., Jia R., Zou Y., Yin L., Li L., He C., Liang X. (2019). Tannic Acid Accelerates Cutaneous Wound Healing in Rats Via Activation of the ERK 1/2 Signaling Pathways. Adv. Wound Care.

[6] Sharma V., Paramasivam G., Vergaelen M., Hoogenboom R., Sundaramurthy A. (2021). Tannic Acid-Stabilized Self-Degrading Temperature-Sensitive Poly(2-n-propyl-2-oxazoline)/Gellan Gum Capsules for Lipase Delivery. ACS Appl. Bio Mater..

[7] Ghasemian M., Kazeminava F., Naseri A., Mohebzadeh S., Abbaszadeh M., Kafil H.S., Ahmadian Z. (2023). Recent progress in tannic acid based approaches as a natural polyphenolic biomaterial for cancer therapy: A review. Biomed. Pharmacother..

[8] Youness R.A., Kamel R., Elkasabgy N., Shao P., Farag M.A. (2021). Recent Advances in Tannic Acid (Gallotannin) Anticancer Activities and Drug Delivery Systems for Efficacy Improvement; A Comprehensive Review. Molecules.

[9] Xiong Y., Li Z., Yang X. (2023). Whey protein-tannic acid conjugate stabilized high internal phase Pickering emulsions: Interfacial stability based on covalent crosslinking. Colloids Surf. A Physicochem. Eng. Asp..

[10] Chen L., Gnanaraj C., Arulselvan P., El-Seedi H., Teng H. (2019). A review on advanced microencapsulation technology to enhance bioavailability of phenolic compounds: Based on its activity in the treatment of Type 2 Diabetes. Trends Food Sci. Technol..

[11] Farha A.K., Yang Q.-Q., Kim G., Li H.-B., Zhu F., Liu H.-Y., Gan R.-Y., Corke H. (2020). Tannins as an alternative to antibiotics. Food Biosci..

[12] Wang Y., Li J., Li B. (2016). Nature-Inspired One-Step Green Procedure for Enhancing the Antibacterial and Antioxidant Behavior of a Chitin Film: Controlled Interfacial Assembly of Tannic Acid onto a Chitin Film. J. Agric. Food Chem..

[13] Guimaraes I., Costa R., Madureira S., Borges S., Oliveira A.L., Pintado M., Baptista-Silva S. (2023). Tannic Acid Tailored-Made Microsystems for Wound Infection. Int. J. Mol. Sci..

[14] Yang W., Duan X., Sun H., Fan X., Wang H., Wang W. (2022). Encapsulation of TA in edible nanofibrous mat improves antioxidant efficiency and their modulation of fatty acids profile in flaxseed oil. Int. J. Food Sci. Technol..

[15] Liang X., Cao K., Li W., Li X., McClements D.J., Hu K. (2021). Tannic acid-fortified zein-pectin nanoparticles: Stability, properties, antioxidant activity, and in vitro digestion. Food Res. Int..

[16] Tao X., Shi H., Cao A., Cai L. (2022). Influence of polyphenol-metal ion-coated ovalbumin/sodium alginate composite nanoparticles on the encapsulation of kaempferol/tannin acid. Int. J. Biol. Macromol..

[17] Wang N., Tian X., Cheng B., Guang S., Xu H. (2022). Calcium alginate/silk fibroin peptide/Bletilla striata polysaccharide blended microspheres loaded with tannic acid for rapid wound healing. Int. J. Biol. Macromol..

[18] Mohammadzadeh V., Mahmoudi E., Ramezani S., Navaeian M., Taheri R.A., Ghorbani M. (2023). Design of a novel tannic acid enriched hemostatic wound dressing based on electrospun polyamide-6/hydroxyethyl cellulose nanofibers. J. Drug Deliv. Sci. Technol..

[19] Sahiner N., Sengel S.B. (2016). Tannic acid decorated poly(methacrylic acid) micro and nanoparticles with controllable tannic acid release and antioxidant properties. Colloids Surf. A Physicochem. Eng. Asp..

[20] Salehipour M., Nikpour S., Rezaei S., Mohammadi S., Rezaei M., Ilbeygi D., Hosseini-Chegeni A., Mogharabi-Manzari M. (2023). Safety of metal–organic framework nanoparticles for biomedical applications: An in vitro toxicity assessment. Inorg. Chem. Commun..

[21] Xie L., Wehling R.L., Ciftci O., Zhang Y. (2017). Formation of complexes between tannic acid with bovine serum albumin, egg ovalbumin and bovine beta-lactoglobulin. Food Res. Int..

[22] Pinto A.F., Nascimento J.M.D., Sobral R., Amorim E.L.C., Silva R.O., Leite A.C.L. (2019). Tannic acid as a precipitating agent of human plasma proteins. Eur. J. Pharm. Sci..

[23] Wang J., Liang X., Du Y., Tang Z., Duan X., Sun Z., Zhao J., Xu W., Wang Y., Tang Y. (2024). Enhancement of oral bioavailability of celastrol by chitosan microencapsulated porous starch carriers. Int. J. Biol. Macromol..

[24] Pasban A., Mousavi S.F., Abdollahi S., Hesarinejad M.A. (2024). Evaluating the potential of soy protein isolate/alginate hydrogel as polyphenolic liposome carrier during gastrointestinal tract: A case study on sumac extract. LWT.

[25] Mohsen S., Bakr M.M., ElDegwy M.A., Abouhussein D.M.N., Fares A.R., ElMeshad A.N. (2024). Pomegranate extract-loaded surfactant-free zein nanoparticles as a promising green approach for hepatic cancer: Optimization and in vitro cytotoxicity. Future J. Pharm. Sci..

[26] Tampucci S., Tofani G., Chetoni P., Di Gangi M., Mezzetta A., Paganini V., Burgalassi S., Pomelli C.S., Monti D. (2022). Sporopollenin Microcapsule: Sunscreen Delivery System with Photoprotective Properties. Pharmaceutics.

[27] Jafari Y., Sabahi H., Rezayan A.H. (2025). Stability and loading properties of Curcumin encapsulated in saffron pollen. Food Chem. Adv..

[28] Zhang Q., Zhang Y., Li X., Cao Q., Ma F., Li Y., Xie D., Ma H., Zhang B., Li X. (2024). Preparation and characteristics of soy protein isolate—Sea buckthorn flavone emulsion and their effects of on quality and heterocyclic amines of roasted mutton granules. Int. J. Biol. Macromol..

[29] Rehman A., Tong Q., Korma S.A., Han W., Karim A., Sharif H.R., Ali A., Yaqoob S., Khalifa S.A., Cacciotti I. (2021). Influence of diverse natural biopolymers on the physicochemical characteristics of borage seed oil-peppermint oil loaded W/O/W nanoemulsions entrapped with lycopene. Nanotechnology.

[30] Castro-Criado D., Jiménez-Rosado M., Perez-Puyana V., Romero A. (2023). Soy Protein Isolate as Emulsifier of Nanoemulsified Beverages: Rheological and Physical Evaluation. Foods.

[31] Chen S., Fan L., Chen X., Guo Z., Zhang B. (2025). Microencapsulation of Lonicera caerulea pomace extract by spray drying: Characterization and stability studies. LWT—Food Sci. Technol..

[32] Chao Song Z., Zhang H., Fei Niu P., Shi L.S., Yan Yang X., Hong Meng Y., Yu Wang X., Gong T., Rong Guo Y. (2023). Fabrication of a novel antioxidant emulsifier through tuning the molecular interaction between soy protein isolates and young apple polyphenols. Food Chem..

[33] Singh I.R., Pulikkal A.K. (2024). Nano emulsions stabilized by natural emulsifiers: A comprehensive review on feasibility, stability and bio-applicability. J. Drug Deliv. Sci. Technol..

[34] Guo Q., Li S., Du G., Chen H., Yan X., Chang S., Yue T., Yuan Y. (2022). Formulation and characterization of microcapsules encapsulating carvacrol using complex coacervation crosslinked with tannic acid. LWT—Food Sci. Technol..

[35] Raj R., Kahraman R., Shakoor A., Montemor F., Taryba M. (2022). Tannic Acid-Loaded Hydroxyapatite Carriers for Corrosion Protection of Polyolefin-Coated Carbon Steel. Appl. Sci..

[36] Heidari F., Akbarzadeh I., Nourouzian D., Mirzaie A., Bakhshandeh H. (2020). Optimization and characterization of tannic acid loaded niosomes for enhanced antibacterial and anti-biofilm activities. Adv. Powder Technol..

[37] Yang G., Huang X., Cai J., Zhang Q. (2021). Curing mechanism of triglycidylamine crosslinked soybean protein adhesive analyzed by Fourier transform infrared, second derivative infrared and two-dimensional correlation spectroscopy. Int. J. Adhes. Adhes..

[38] Hu W., Chen C., Wang Y., He W., He Z., Chen J., Li Z., Li J., Li W. (2023). Development of high internal phase emulsions with noncovalent crosslink of soy protein isolate and tannic acid: Mechanism and application for 3D printing. Food Chem..

[39] Nam S., Easson M.W., Condon B.D., Hillyer M.B., Sun L., Xia Z., Nagarajan R. (2019). A reinforced thermal barrier coat of a Na–tannic acid complex from the view of thermal kinetics. RSC Adv..

[40] Hadzieva J., Mladenovska K., Simonoska Crcarevska M., Glavaš Dodov M., Dimchevska S., Geškovski N., Grozdanov A., Popovski E., Petruševski G., Chachorovska M. (2017). Lactobacillus casei loaded Soy Protein-Alginate Microparticles prepared by Spray-Drying. Food Technol. Biotechnol..

[41] Jiang K., Lei Z., Yi M., Lv W., Jing M., Feng Q., Tan H., Chen Y., Xiao H. (2021). Improved performance of soy protein adhesive with melamine–urea–formaldehyde prepolymer. RSC Adv..

[42] Boachie R.T., Okagu O.D., Abioye R., Huttmann N., Oliviero T., Capuano E., Fogliano V., Udenigwe C.C. (2022). Lentil Protein and Tannic Acid Interaction Limits in Vitro Peptic Hydrolysis and Alters Peptidomic Profiles of the Proteins. J. Agric. Food Chem..

[43] Omar A., Arken A., Wali A., Gao Y., Aisa H.A., Yili A. (2022). Effect of phenolic compound-protein covalent conjugation on the physicochemical, anti-inflammatory, and antioxidant activities of silk sericin. Process Biochem..

[44] Pi X., Liu J., Sun Y., Ban Q., Cheng J., Guo M. (2022). Protein modification, IgE binding capacity, and functional properties of soybean protein upon conjugation with polyphenols. Food Chem..

[45] Wu J., Ding X., Zhang J., Chen W. (2018). Online Determination of Colloidal Properties of Tannin Solutions under Microwave Irradiation using a Modified Zetasizer. Anal. Lett..

[46] Poncet-Legrand C., Gautier C., Cheynier V., Imberty A. (2007). Interactions between flavan-3-ols and poly(L-proline) studied by isothermal titration calorimetry: Effect of the tannin structure. J. Agric. Food Chem..

[47] Zhao Y., Xu L., Kong F., Yu L. (2021). Design and preparation of poly(tannic acid) nanoparticles with intrinsic fluorescence: A sensitive detector of picric acid. Chem. Eng. J..

[48] Liang X., Cheng W., Liang Z., Zhan Y., McClements D.J., Hu K. (2022). Co-Encapsulation of Tannic Acid and Resveratrol in Zein/Pectin Nanoparticles: Stability, Antioxidant Activity, and Bioaccessibility. Foods.

[49] Zhang A., Chen S., Wang Y., Wang X., Xu N., Jiang L. (2020). Stability and in vitro digestion simulation of soy protein isolate-vitamin D3 nanocomposites. LWT.

[50] Oppermann A.K.L., Noppers J.M.E., Stieger M., Scholten E. (2018). Effect of outer water phase composition on oil droplet size and yield of (w(1)/o/w(2)) double emulsions. Food Res. Int..

[51] Neumann S.M., Scherbej I., van der Schaaf U.S., Karbstein H.P. (2018). Investigations on the influence of osmotic active substances on the structure of water in oil emulsions for the application as inner phase in double emulsions. Colloids Surf. A Physicochem. Eng. Asp..

[52] Zhang X., Xu W., Li X., Pan G., Chen N., Xie Q., Wang X. (2023). Preparation of pH Sensitive Bacteriostatic W/O/W Emulsion Microcapsules. J. Biomater. Sci. Polym. Ed..

[53] Zong X., Li H., Tang Q., Wang X., Li Y., Li L. (2022). Preparation and characterization of glucoamylase microcapsules prepared by W/O/W type complex coacervation freeze drying. J. Food Sci..

[54] Ozturk A.A., Namli I., Gulec K., Kiyan H.T. (2020). Diclofenac sodium loaded PLGA nanoparticles for inflammatory diseases with high anti-inflammatory properties at low dose: Formulation, characterization and in vivo HET-CAM analysis. Microvasc. Res..

[55] Campos P., Benevenuto C.G., Calixto L.S., Melo M.O., Pereira K.C., Gaspar L.R. (2019). Spirulina, Palmaria Palmata, Cichorium Intybus, and Medicago Sativa extracts in cosmetic formulations: An integrated approach of in vitro toxicity and in vivo acceptability studies. Cutan. Ocul. Toxicol..

[56] Zhang S., Ye T. (2021). Preparation of Natural Composite Microcapsules Containing Orchid Black Currant Fragrance and its Sustained-Release Properties on Hair Bundle. J. Polym. Environ..

[57] Yu N., Luo Z., Ma F., Li J., Yang P., Li G., Li J. (2023). Cationic Gelatin Cross-Linked with Transglutaminase and Its Electrospinning in Aqueous Solution. Langmuir.

[58] Dong S., Hu S.-M., Yu S.-J., Zhou S., Zhou T. (2023). Soybean protein isolate/chitosan complex-rutin microcapsules. Int. J. Biol. Macromol..

[59] Yang S., Li X., Zhang H. (2024). Ultrasound-assisted extraction and antioxidant activity of polysaccharides from *Tenebrio molitor*. Sci. Rep..

